# Big End Double-Layer Stents for the Treatment of Gastric Outlet Obstruction Caused by Stomach Cancer

**DOI:** 10.1155/2019/8093091

**Published:** 2019-07-01

**Authors:** Ding Shi, Dong Wu, Yongpan Liu, Feng Ji, Yinsu Bao

**Affiliations:** ^1^Department of Gastroenterology, Huamei Hospital, University of Chinese Academy of Sciences, Ningbo 315010, China; ^2^Department of Gastroenterology, The First People's Hospital of Yuhang District, Hangzhou 311100, China; ^3^Department of Gastroenterology, The First Affiliated Hospital of Zhejiang University, Hangzhou 310003, China; ^4^Department of Gastroenterology, The First Affiliated Hospital of Henan University of Chinese Medicine, Zhengzhou 450000, China

## Abstract

**Objectives:**

This study is aimed at evaluating the efficacy and safety of the big end double-layer uncovered self-expanding metal stents (SEMS) for the treatment of gastric outlet obstruction (GOO) caused by distal stomach cancer.

**Methods:**

Seventy three patients receiving big end double-layer uncovered SEMS for the treatment of GOO caused by distal gastric cancer will be included in this multicenter prospective clinical trial. The main outcome measures included the functional outcome, the complications, the reinterventional rates, the average treatment charges, and the mean survival time. Monthly telephone calls were needed to assess the food intake until the patients died.

**Results:**

The technical and the clinical success rates were 98.6%. The stent obstruction caused by tumor ingrowth was observed in one patient (1.4%). The incidence of food impaction was 2.9% (2/70) and the reinterventional rate was 4.3% (3/70). However, stent migration and obstruction caused by overgrowth were not observed. No perforation and severe bleeding were observed. The median cost of endoscopic stenting and total hospitalization (including reinterventions) for the big end double-layer uncovered SEMS in this study was $2945 and $3408, respectively. The mean survival time was 212.5 days.

**Conclusions:**

The placement of big end double-layer uncovered SEMS is a safe and effective modality and has the potential to be one of the options for the treatment of GOO caused by the distal gastric cancer.

## 1. Introduction

Most malignant GOO occurred as a preterminal adverse event in advanced gastric carcinoma, which cannot be radically treated by surgery. About 50% of the patients with GOO presented with nausea, vomiting, and decreasing oral intake [1]. For decades, SEMS have become one of the best treatments for GOO [2–4]. Previous studies demonstrated that stent placement could improve the life quality for GOO patients [5] and provide faster symptom relief ^[^6^]^. However, higher migration and reobstruction rates are major defects of standard covered and uncovered SEMS [5, 7–9]. Therefore, considering no stent was specific designed for the treatment of GOO caused by stomach cancer; how to reduce the incidents of stent migration and restenosis is an important clinical problem. In our previous studies, the big funnel covered SEMS were designed according to the sizes of the proximal end of GOO. The designed stents had excellent lesion coverage and shaping effect and were superior to other stents in terms of survival time, cost of stent treatment, and preventing stent migration or reobstruction [10, 11]. However, the big funnel and individualized covered SEMS cannot be implanted by endoscopic channel (through-the-scope (TTS)). If the big end stent was preinstalled in the delivery system with smaller outer diameter, it could be implanted through TTS. Our previous tests showed that TTS delivery system could accommodate a stent with a maximum end of 40 mm in diameter, but could not accommodate the same covered stent. How to combine the advantages of big end covered stent and TTS implantation is an urgent clinical practice problem. Will the coaxial implantation of a big end uncoated stent and a standard uncoated stent achieve the same clinical effects as a big end covered stent? We hypothesized that the big end double-layer uncovered SEMS could be used for GOO caused by distal gastric cancer and overcame the shortcomings, such as tumor ingrowth with uncovered SEMS and migration with covered SEMS and failed implantation by TTS. In the current study, we investigated the efficacy and safety of the big end double-layer uncovered SEMS for the treatment of unresectable distal gastric cancer with GOO, focusing on technical success, clinical success, adverse events, reinterventional rates, average treatment charges, and survival time.

## 2. Methods

This study was designed as a multicenter, prospective clinical trial. The participating hospitals were the Ningbo No. 2 Hospital, the First Affiliated Hospital of Zhejiang University, the First Affiliated Hospital of Henan University of Chinese Medicine, and the First People's Hospital of Yuhang District. This study was conducted from March 2006 to March 2018 and approved by the ethics committees of the four involved hospitals and with all the relevant patients' informed consent.

### 2.1. Patients

The inclusion criteria of patient were as follows: (1) with gastric cancer, (2) with symptomatic malignant GOO, and (3) with malignant obstruction located between the gastric body and duodenal bulb. Meanwhile, the patients with one of the following symptoms must be excluded from the study: (1) mild symptoms of liquid oral intake; (2) candidates for surgery; (3) gastrointestinal perforation, another intestinal obstruction origin, or diabetes, or used promotility drugs; or (4) severe comorbidities that were contraindications for stenting procedure.

Esophagogastroduodenoscopy was done and GOO score was estimated according to the method reported by Song et al. [12]: 0, eat a normal diet; 1, eat solid food; 2, eat soft food; 3, swallow liquids only; 4, no oral intake without vomiting; and 5, no oral intake with vomiting.

### 2.2. Stent Design

**Figure 1:**
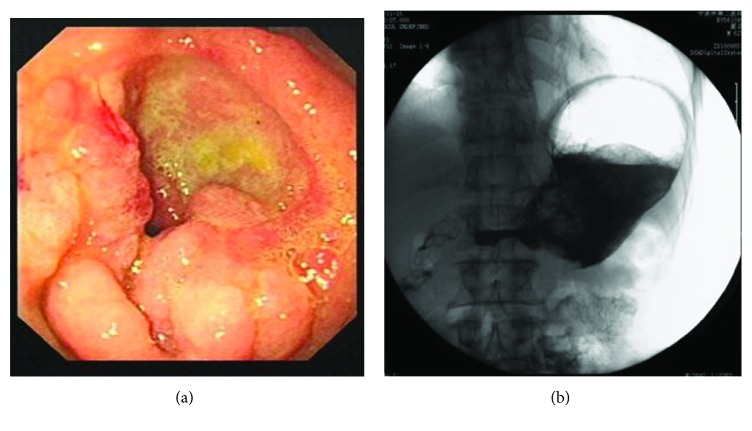
One example of shapes in proximal lumens of GOO. (a) Endoscopic view of a tumor in the distal gastric cavity. (b) The shape of the proximal lumen in the GOO.

**Figure 2:**
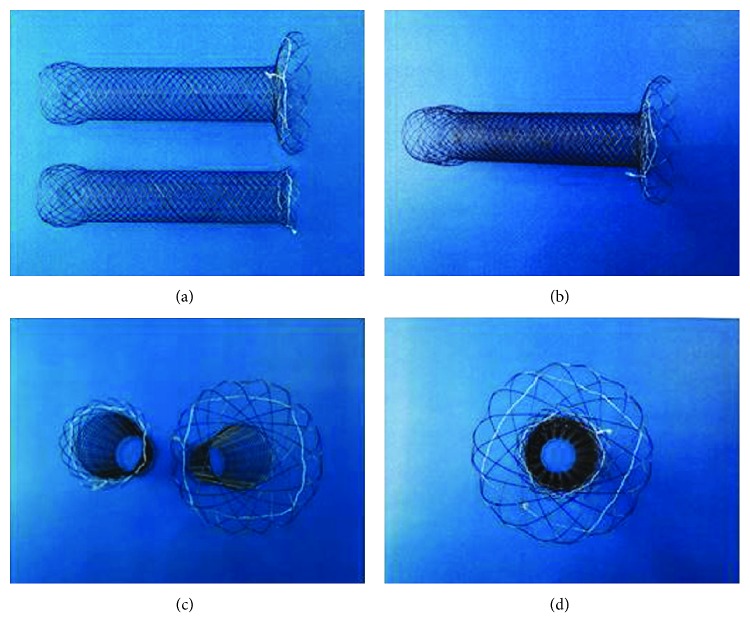
The photographs of the inner, outer, and combined two stents: (a, c) the inner and outer stents and (b, d) the combined stent.

The proximal end of GOO was observed by gastroscopy (Figure 1(a)) and radiography (Figure 1(b)). Upper gastrointestinal radiography was performed within three days before the stent design. The big end double-layer uncovered stent was made of 2 braided nitinol stents. The outer stent was a big end uncovered stent (custom made, price $949 per stent, Micro-Tech (Nanjing) Co., Ltd., Nanjing, Jiangsu, China), which was designed with a cup opening of 40 mm in diameter and 10 mm in length. The distal part of all stents was semispherical, with a length of 20 mm and a diameter of 26 mm (Figures 2(a) and 2(c)). The body of the stent was 20 mm in diameter and 100 mm in the overall length. The stents were mounted on a delivery system with an outer diameter of 3.5 mm and an overall length of 130 to 180 cm. The inner stent was a standard uncovered stent MTN-CG-s-20/80 (Micro-Tech (Nanjing) Co., Ltd., Nanjing, Jiangsu, China), the end of which was semispherical with a diameter of 26 mm and a length of 20 mm (Figures 2(a) and 2(c)). The length of the inner stent was 80 mm. The combined two stents are showed in Figures 2(b) and 2(d).

The following information was recorded: patient demographics, comorbidities, abdominal pain related to the procedure, hemorrhage, perforation, technical and clinical success, coverage rate of the stent cup over the proximal lesion (whether the lesions in the residual antral cavity of GOO were completely covered by the stent cup, to be checked by the endoscopy), shaping effect (whether the stent cup was fit for the proximal residual antral cavity or not), GOOSS, stent migration, food impaction, reobstruction of stent by tumor ingrowth or overgrowth, and patient survival time.

### 2.3. Procedures

**Figure 3:**
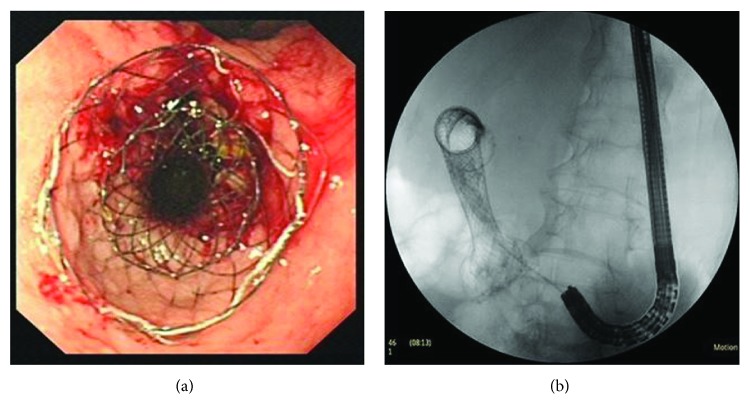
The use of a big end double-layer stent for the treatment of GOO. (a) Endoscopic view of the proximal big end double-layer stent at the pyloric area. (b) Confirmation of stent deployment by fluoroscopy.

The big end double-layer SEMS were implanted by a through-the-scope method. The endoscope with a working channel of 3.7 mm (GIF-2T200, Olympus, Tokyo, Japan) or a therapeutic duodenoscope with a working channel of 4.2 mm (ED-250XT5, Fujinon, or TJF 240, Olympus) was used. Under fluoroscopic guidance, the ERCP guide wire was placed distal to the stenosis and confirmed to be located in the intestinal lumen by radiography. Then, the delivery system (MTN-CR-3.3/160, Micro-Tech (Nanjing) Co., Ltd., Nanjing, Jiangsu, China) was passed over through an ERCP guide wire by two steps. Briefly, a big end stent was put in with the guidance the endoscopic and the fluoroscopic. Before half of the stent was released, its position could be adjusted at any time to allow the big cup end to cover the edge of the proximal lesion of the GOO and then implant the inner stent in the same way. After the stent was released completely, the endoscopic and fluoroscopic views were obtained to confirm the position of the stent (Figures 3(a) and 3(b)).

### 2.4. Follow-Up

After 1 to 3 day intervention, abdominal radiograph was performed to check stent expansion and location. The evaluation of GOO scores was required 7 and 14 days after the stent placement. Monthly telephone calls were needed to assess the food intake until the patients died. In some cases, the follow-up data were obtained from the patients' family via an interview at the interval of one month with a doctor who was responsible for the patients' condition. If nausea and vomiting were reported, the patient would be inspected by endoscopy or radiography to explore the etiological factor, since they could be caused by GOO recurrence or stent migration or both of them.

### 2.5. Outcome Measurements

Stent migration and reobstruction rate were the primary outcomes. The secondary outcomes based on the GOO scoring system (GOOSS) were as follows: coverage rate of the stent cup over the proximal lesion, shaping effect (stent cup fit into the residual antral cavity, i.e., endoscopy showed that the lesions in the residual antral cavity of GOO were completely covered by the stent cup), technical and clinical success, complications related to the procedure (e.g., hemorrhage, perforation), and survival time. Technical success was defined as the success of an appropriate placement of the SEMS across the stenosis, which was confirmed by a combination of endoscopy and fluoroscopy. Clinical success was determined by the resolution of obstructive symptoms and the ability to restart a low diet after stent placement.

## 3. Statistical Analysis

Statistical analyses were performed using SPSS for Windows (version 22.0, Chicago, SPSS Inc.). Continuous variables were expressed as mean standard deviation (SD). Categorical data were expressed as *n* (the number of cases) or %. The paired *t*-test was used to compare the GOOSS score before and after stent implantation. The *P* value <0.05 was considered to be statistically significant.

## 4. Results

### 4.1. Patient Characteristics

Patient characteristics are showed in Table 1. In this study, the whole stricture segment was traversed by one big end double-layer uncovered stent in all patients and no additional stent was used.

### 4.2. Technical and Clinical Outcomes

The efficacy and complication are shown in Table 2. One stent could not be implanted successfully since the guide wire could not pass through the narrow lesions. There was one case after stent placement showed no improvement in symptoms. The other patients showed improvements of 2 grades in the level of dietary intake in the second week after stent placement. The GOOSS score after stent implantation was significantly improved compared with that before stent implantation (*P* < 0.05).

### 4.3. Stent Complications

Stent obstruction caused by tumor ingrowth was observed in one patient during the follow-up period. The developed stent obstruction was found after 365 days. The incidence of food impaction was 2.9%, which was treated endoscopically after stent placement. In case of restenosis, a standard uncovered stent was reinserted to overlap the primary stent. Stent migration and obstruction caused by tumor overgrowth were not found in the present study.

Two cases of bleeding and six cases of abdominal pain occurred in the study, but they were mild and did not need special treatment. No perforation and severe bleeding were observed.

### 4.4. Survival Time

During the follow-up period after stent placement, 70 patients died. The mean survival time was 212.5 days. Two patients did not come to the hospital for follow-up.

## 5. Discussion

GOOs caused by distal gastric cancer are usually wide at the top and narrow at the bottom. The range of lesions in the proximal GOO is often widespread. Standard stents often fail to fully cover lesions in the proximal GOO and effectively eliminate stent migration and obstruction caused by overgrowth due to their smaller proximal ends [13–18]. Therefore, it is necessary to increase diameters of proximal stents' ends. The individualized stent could help reduce tumor ingrowth by covering the stent body with a membrane and stent migration by increasing the diameter of both stents' ends [19]. However, the special stent could not be implanted by means of TTS, which is not conducive to clinical promotion. In this study, we tried to solve the problem of big end SEMS implantation by coaxial implantation of an uncovered stent with a proximal end of 40 mm in diameter and a conventional uncovered stent through TTS. The present study demonstrated that the big end double-layer uncovered SEMS had a good performance in preventing stent obstruction and migration. The incidence of stent obstruction, food impaction, and reinterventional rate was 1.4%, 2.9%, and 4.3%, respectively, which were similar to the individualized and funnel covered SEMS^[^10^,^^19^^]^, but much lower compared with other reports [20, 21]. Unlike the individualized and funnel SEMS [10, 19], neither distal nor proximal migration of the big end double-layer SEMS was not found in this study. In addition, the mean survival time of patients in this study was longer than those in other reports [22, 23]. We speculated that double-layer uncovered SEMS could significantly delay tumor ingrowth by reducing its mesh size and the larger proximal ends provided a wider space to accommodate tumor ingrowth and slowed stent obstruction caused by tumor overgrowth. At the same time, the double-layer stent could reduce the collapse of the stent since its double-layer structure could provide increased support force. The low migration rate is a common advantage of uncovered stent, which was used in this study. Therefore, the factors mentioned above might be the reason for lower reintegration rate and longer survival time in this study. Moreover, the improvement of GOOSS score was obvious after stent implantation, which mainly depended on the shaping effect of the big cup of stent and the coverage of the proximal obstructive lesions by the big cup of stent. Because the big end stent was not only fitting well in the remnant stomach cavity but also providing a good pathway for the passage of food [10, 19], only two cases of food obstruction occurred in this study, which might be related to the food adhesion caused by the nonsmooth wall of the uncovered stents.

The mean cost of treatment with the big end double-layer uncovered SEMS was $2,945, which was higher than that with the individualized or funnel SEMS in China [10, 19]. Although the cost of the big end double-layer uncovered SEMS was higher, it was cost-efficient for the long-term run due to its lower reintervention rate (4.3%) and longer survival time (212.5 days) compared with monolayer SEMS [24–27]. Thus, the big end double-layer uncovered SEMS did not increase the total treatment cost for patients with GOO.

Generally, the big end double-layer uncovered stent not only does have the advantage of large end to reduce the tumor overgrowth but also reduces tumor ingrowth due to its double-layer effect and prevents stent migration (advantage of uncovered SEMS). Therefore, it has the potential to be one of the options for the treatment of GOO caused by the distal gastric cancer.

The limitation of this study was that this study was not a randomized controlled trial so we could not accurately judge whether the big end double-layer uncovered stent was superior to the other ones. Further study is still needed to compare the big end double-layer uncovered stent with other SEMS.

In conclusion, the placement of big end double-layer uncovered SEMS is a safe and effective modality and has the potential to be one of the options for the treatment of GOO caused by the distal gastric cancer.

## Figures and Tables

**Table 1:** Patient characteristics.

Characteristic	No.
Male/female	36/37
Average age	78.8
Comorbidities	19/73
Differentiated degree	
Moderately	18
Poorly	55
TNM staging	
III	19
IV	54
Chemotherapy	5/73
Cup/funnel shape	51/22
GOOSS (before stent)	4.4 ± 0.5

TNM = tumor, node, metastasis; GOOSS = gastric outlet obstruction score system.

**Table 2:** Efficacy and complications.

Characteristic	No.
Technical success	98.6% (72/73)
Clinical success	98.6% (71/72)
Covering lesion rate	97.2% (70/72)
Shaping effect	97.2% (70/72)
GOOSS (after stent)	2.0 ± 0.5
Stent obstruction	
Ingrowth	1.4% (1/70)
Overgrowth	0% (0/70)
Food impaction	2.9% (2/70)
Migration	0% (0/70)
Reintervention	4.3% (8/70)
Bleeding	2.8% (2/72)
Abdominal pain	4.5% (6/72)
Costs	
Endoscopic stenting	2945
Total hospital (IR)	3228
Survival (days)	212.5

GOOSS = gastric outlet obstruction score system; IR = including reinterventions.

## Data Availability

The data used to support the findings of this study are restricted by the Ethics Committees of the Ningbo No. 2 Hospital, the First Affiliated Hospital of Zhejiang University, the First Affiliated Hospital of Henan University of Chinese Medicine, and the First People's Hospital of Yuhang District in order to protect patient privacy.
